# Supplementary material for: A unique mating strategy without physical contact during fertilization in Bombay Night Frogs (Nyctibatrachus humayuni) with the description of a new form of amplexus and female call
Source: PeerJ. 2016 Jun 14;4:e2117. doi: 10.7717/peerj.2117 (PMC4911947; doi:10.7717/peerj.2117)
Supplement: Supplemental Information 14 — (A) Distribution of N. humayuni in the Western Ghats of India. Study site is marked in red. (B) A mountain stream flowing over laterite rocks with overhanging vegetation, which is the typical breeding habitat of N. humayuni in the forest at Humbarli village, Koyna. (C) An adult male of N. humayuni is seen calling from overhanging vegetation. [file peerj-04-2117-s014.pdf]

Supplemental Information

Figure S1

Bert Willaert, Robin Suyesh, Sonali Garg, Varad B Giri, Mark A Bee and SD Biju

A unique mating strategy without physical contact during fertilization in Bombay Night Frog (*Nyctibatrachus humayuni*) with the description of a new form of amplexus and female call

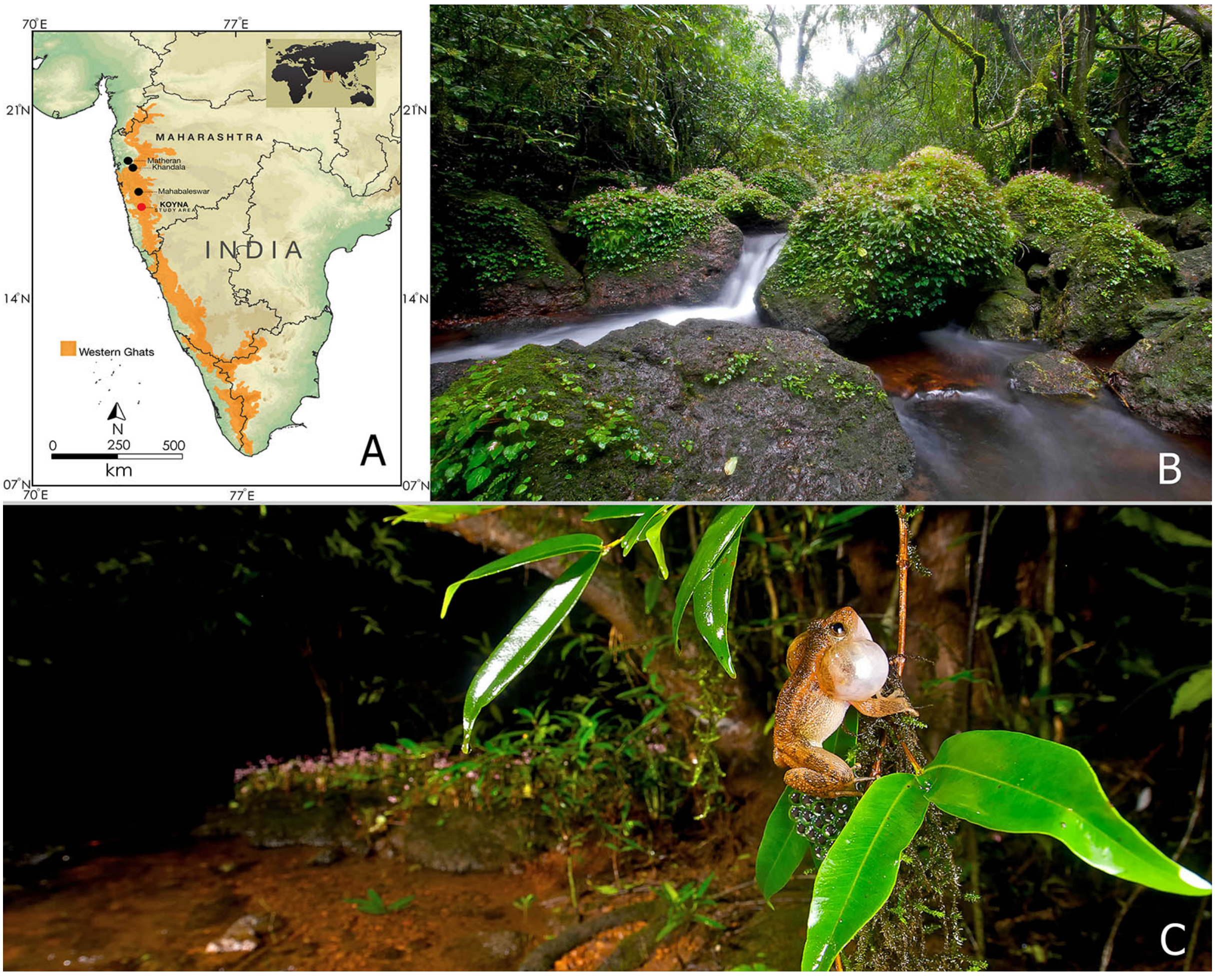

Figure S1 Study site and breeding habitat of *Nyctibatrachus humayuni*. (A) Distribution of *N. humayuni* in the Western Ghats of India. Study site is marked in red. (B) A mountain stream flowing over laterite rocks with overhanging vegetation, which is the typical breeding habitat of *N. humayuni* in the forest at Humbarli village, Koyna. (C) An adult male of *N. humayuni* is seen calling from overhanging vegetation.
