# Supplementary material for: A unique mating strategy without physical contact during fertilization in Bombay Night Frogs (Nyctibatrachus humayuni) with the description of a new form of amplexus and female call
Source: PeerJ. 2016 Jun 14;4:e2117. doi: 10.7717/peerj.2117 (PMC4911947; doi:10.7717/peerj.2117)
Supplement: Supplemental Information 15 — (A) Male calling from a fallen tree trunk. (B) Ventral view showing bright orange coloured femoral glands, indicated by an arrow. [file peerj-04-2117-s015.pdf]

Supplemental Information

Figure S2

Bert Willaert, Robin Suyesh, Sonali Garg, Varad B Giri, Mark A Bee and SD Biju

A unique mating strategy without physical contact during fertilization in Bombay Night Frog (*Nyctibatrachus humayuni*) with the description of a new form of amplexus and female call

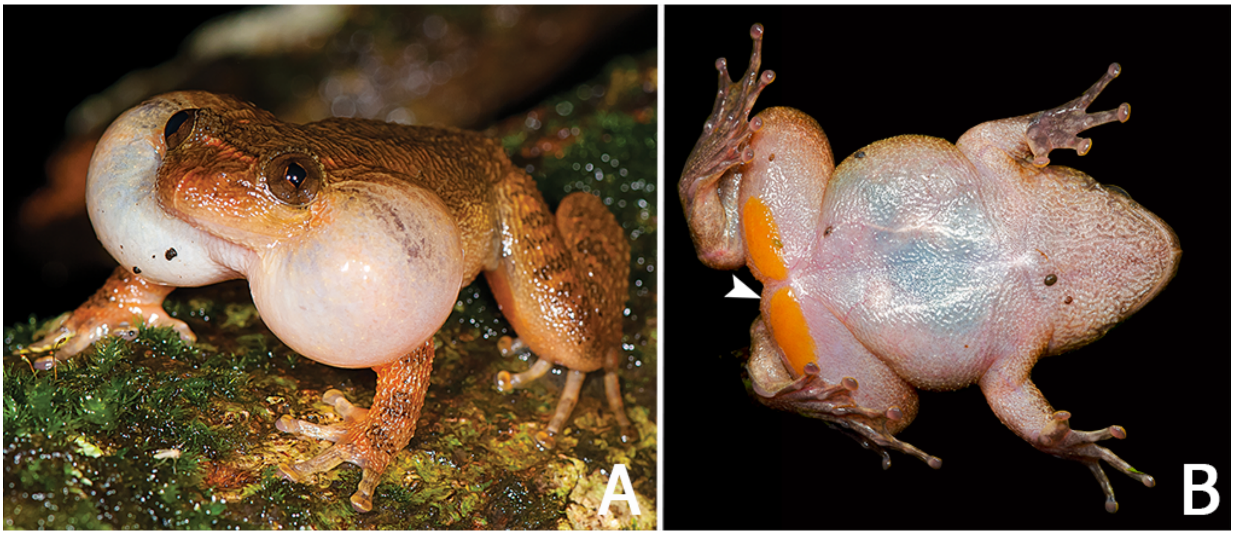

Figure S2 An adult male of *Nyctibatrachus humayuni* (in life). (A) Male calling from a fallen tree trunk. (B) Ventral view showing bright orange coloured femoral glands, indicated by an arrow.
