# Supplementary material for: A unique mating strategy without physical contact during fertilization in Bombay Night Frogs (Nyctibatrachus humayuni) with the description of a new form of amplexus and female call
Source: PeerJ. 2016 Jun 14;4:e2117. doi: 10.7717/peerj.2117 (PMC4911947; doi:10.7717/peerj.2117)
Supplement: Supplemental Information 16 — Properties analyzed are after Bee, Suyesh & Biju (2013a) and Bee, Suyesh & Biju (2013b). [file peerj-04-2117-s016.doc]

Supplemental Information: Table S1

Bert Willaert, Robin Suyesh, Sonali Garg, Varad B Giri, Mark A Bee and SD Biju

A unique mating strategy without physical contact during fertilization in Bombay Night Frog (*Nyctibatrachus humayuni*) with the description of a new form of amplexus and female call

**Table S1 Description of the different properties that are used to describe the male and female calls.** Properties analyzed are after *Bee, Suyesh & Biju, 2013a* and *Bee, Suyesh & Biju, 2013b*.

| **Call Properties** | **Description** |
| --- | --- |
| **A. Male** |  |
| *Overall call properties* |  |
| Call duration (ms) | Time between onset of non-pulsatile part and offset of last pulse in a call |
| Call rise time (ms) | Time between onset of non-pulsatile part and point of maximum amplitude |
| Call fall time (ms) | Time between point of maximum amplitude and offset of last pulse |
| Overall Dominant frequency (kHz) | Maximum frequency using Raven’s selection spectrum function over the duration of the entire call |
| *Properties of 1^st^ part of the call* |  |
| Duration 1st part (ms) | Time between onset of non-pulsatile part and onset of pulsatile part |
| Rise Time (ms) | Time between onset of non-pulsatile part and point of maximum amplitude in 1st part |
| Fall Time (ms) | Time between point of maximum amplitude in 1st part and offset of non-pulsatile part |
| Overall Dominant Frequency (Hz) | Maximum frequency using Raven’s selection spectrum function over the duration of non-pulsatile part |
| Dominant Frequency 1 (Hz) | Maximum frequency using Raven’s selection spectrum function over the 1st quarter of non-pulsatile part |
| Dominant Frequency 2 (Hz) | Maximum frequency using Raven’s selection spectrum function over the 2nd quarter of non-pulsatile part |
| Dominant Frequency 3 (Hz) | Maximum frequency using Raven’s selection spectrum function over the 3rd quarter of non-pulsatile part |
| Dominant Frequency 4 (Hz) | Maximum frequency using Raven’s selection spectrum function over the 4th quarter of non-pulsatile part |
| *Properties of 2^nd^ part of the call* |  |
| Duration 2nd part (ms) | Time between onset of pulsatile part and offset of last pulse in a call |
| Rise Time (ms) | Time between onset of pulsatile part and point of maximum amplitude in 2nd part |
| Fall Time (ms) | Time between point of maximum amplitude in 2nd part and offset of last pulse in a call |
| # Pulses per Call (2nd part) | Count of pulses (k) |
| Pulse Rate (pulses/s) 2nd part | Number of pulses minus 1 (k-1), divided by time between onset of first pulse and onset of last pulse |
| First Pulse Period (ms) | Time between first pulse onset to onset of next pulse |
| Middle Pulse Period (ms) | Time between middle pulse onset to onset of next pulse |
| "N-1" Pulse Period (ms) | Time between second last pulse onset to onset of last pulse |
| Overall Dominant Frequency 2nd part (Hz) | Maximum frequency using Raven’s selection spectrum function over the duration of pulsatile part |
| First Pulse Dominant Frequency (Hz) | Maximum frequency using Raven’s selection spectrum function over the first pulse of 2nd part |
| Middle Pulse Dominant Frequency (Hz) | Maximum frequency using Raven’s selection spectrum function over the middle pulse of 2nd part |
| Last Pulse Dominant Frequency (Hz) | Maximum frequency using Raven’s selection spectrum function over the last pulse of 2nd part |
| *Properties of pulse of maximum amplitude* | |
| Time of Pulse (ms) from the beginning of call | Time between onset of non-pulsatile part and onset of maximum amplitude pulse |
| Pulse Period (ms) | Time between pulse onset to onset of next pulse |
| Pulse Duration (ms) | Time between pulse onset and offset (If pulse duty cycle ¼ 100%, then pulse duration equivalent to pulse period.) |
| Call fall time (ms) | Time between point of maximum amplitude and offset of last pulse |
| Pulse rise time | Time between the onset of the pulse (maximum amplitude pulse) and the point where it attains its maximum amplitude |
| Pulse 50% rise time | Time between the onset of the pulse (maximum amplitude pulse) and the point where it attains 50% of its maximum amplitude |
| Pulse fall time | Time between the point of maximum amplitude and the offset of the pulse (maximum amplitude pulse) |
| Pulse 50% fall time | Time between the point of 50% of maximum amplitude and the offset of the pulse (maximum amplitude pulse) |
| **B. Female** |  |
| Call duration (ms) | Time between onset and offset of a call |
| Call rise time (ms) | Time between onset of call and point of maximum amplitude |
| Call 50% rise time | Time between the onset of the call and the point where it attains 50 % of its maximum amplitude |
| Call fall time (ms) | Time between point of maximum amplitude and offset of call |
| Call 50% fall time | Time between the point of 50 % of maximum amplitude and the offset of the call |
| Overall Dominant frequency (kHz) | Maximum frequency using Raven’s selection spectrum function over the duration of the entire call |
| Dominant Frequency 1 (Hz) | Maximum frequency using Raven’s selection spectrum function over the first frequency peak |
| Dominant Frequency 2 (Hz) | Maximum frequency using Raven’s selection spectrum function over the second frequency peak |
| Dominant Frequency 3 (Hz) | Maximum frequency using Raven’s selection spectrum function over the third frequency peak |
