# Supplementary material for: A unique mating strategy without physical contact during fertilization in Bombay Night Frogs (Nyctibatrachus humayuni) with the description of a new form of amplexus and female call
Source: PeerJ. 2016 Jun 14;4:e2117. doi: 10.7717/peerj.2117 (PMC4911947; doi:10.7717/peerj.2117)
Supplement: Supplemental Information 17 — Shown here are the means (X), standard deviation (SD) and range of individual means (paranthesis include minimum and maximum values across all analyzed calls). CVs computed both between individuals (CVb) and within individuals (CVw). [file peerj-04-2117-s017.doc]

Supplemental Information: Table S2

Bert Willaert, Robin Suyesh, Sonali Garg, Varad B Giri, Mark A Bee and SD Biju

A unique mating strategy without physical contact during fertilization in Bombay Night Frog (*Nyctibatrachus humayuni*) with the description of a new form of amplexus and female call

**Table S2 Overview of descriptive statistics of male *Nyctibatrachus humayuni* advertisement calls, based on the values determined from a sample of eight individuals.** Shown here are the means (
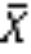
), standard deviation (SD) and range of individual means (paranthesis include minimum and maximum values across all analyzed calls). CVs computed both between individuals (CVb) and within individuals (CVw).

| **Type of acoustic properties** | **Property** | 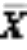 | **SD** | **Range (min-max)** | **CV_b_** | **Mean CV_w_ (range)** |
| --- | --- | --- | --- | --- | --- | --- |
| **A. Entire call** |  |  |  |  |  |  |
| Temporal call properties | Call Duration (ms) | 532.24 | 54.80 | 482.80–630.05 (382.62–693.43) | 10.30 | 7.40 (4.28–13.38) |
|  | Call Rise Time (ms) | 267.63 | 53.66 | 189.40–367.67 (120.04–434.28) | 20.05 | 12.84 (5.33–26.22) |
|  | Call Fall Time (ms) | 262.45 | 33.02 | 215.15–305.02 (123.23–361.79) | 12.58 | 11.09 (6.18–19.58) |
| Spectral property | Overall Dominant Frequency (kHz) | 1.34 | 0.11 | 1.11–1.45 (1.03–1.55) | 8.19 | 3.73 (2.15–5.85) |
| **B. First part of call** |  |  |  |  |  |  |
| Temporal call properties | Duration 1st part (ms) | 288.86 | 58.63 | 212.27–385.134 (143.18–440.0) | 20.30 | 11.74 (5.40–21.71) |
|  | Rise Time 1st part (ms) | 265.18 | 54.09 | 188.82–364.56 (120.04–434.28) | 20.40 | 13.06 (5.33–25.55) |
|  | Fall Time 1st part (ms) | 23.61 | 12.30 | 9.21–43.77 (4.78–81.85) | 52.10 | 50.65 (40.17–68.13) |
| Spectral properties | Overall Dominant Frequency (kHz) | 1.34 | 0.11 | 1.11–1.45 (1.03–1.55) | 8.16 | 3.73 (2.15–5.85) |
|  | Dominant Frequency 1 (kHz) | 1.03 | 0.08 | 0.91–1.15 (0.86–1.20) | 7.34 | 2.51 (1.45–5.25) |
|  | Dominant Frequency 2 (kHz) | 1.12 | 0.09 | 0.96–1.23 (0.90–1.29) | 7.64 | 2.97 (1.98–5.84) |
|  | Dominant Frequency 3 (kHz) | 1.23 | 0.10 | 1.04–1.35 (0.94–1.46) | 8.16 | 3.95 (1.76–5.60) |
|  | Dominant Frequency 4 (kHz) | 1.34 | 0.11 | 1.11–1.45 (1.03–1.55) | 8.19 | 3.70 (2.15–5.85) |
| **C. Second part of call** |  |  |  |  |  |  |
| Temporal call properties | Duration 2nd part (ms) | 243.54 | 34.99 | 193.42–284.92 (152.49–332.86) | 14.37 | 8.86 (6.63–14.49) |
|  | Rise Time (ms) | 7.76 | 1.72 | 5.94–10.52 (2.99–48.33) | 22.21 | 73.77 (55.85–99.80) |
|  | Fall Time (ms) | 229.14 | 35.80 | 182.71-268.71 (117.3-312.51) | 15.63 | 10.95 (7.80–18.28) |
|  | # Pulses per Call (2nd part)^*^ | 20.00 | 17.25–22.5 | 16–24 (13–28) | 12.99 | 9.27 (6.44–12.60) |
|  | Pulse Rate (pulses/s) 2nd part | 80.76 | 3.38 | 74.47–84.11 (68.13–87.74) | 4.18 | 2.24 (1.02–4.51) |
|  | First Pulse Period (ms) | 13.08 | 0.79 | 11.88–14.30 (9.43–16.09) | 6.01 | 7.27 (6.27–9.44) |
|  | Middle Pulse Period (ms) | 12.21 | 0.76 | 11.45–13.90 (10.18–25.16) | 6.23 | 6.70 (2.32–27.40) |
|  | "N-1" Pulse Period (ms) | 13.16 | 0.66 | 11.99–13.91 (5.37–19.14) | 5.01 | 8.34 (4.22–24.26) |
| Spectral call properties | Overall Dominant Frequency 2nd part (kHz) | 1.38 | 0.11 | 1.18–1.52 (1.07–1.59) | 7.89 | 3.75 (2.37–6.73) |
|  | First Pulse Dominant Frequency (kHz) | 1.39 | 0.11 | 1.17–1.53 (1.07–1.59) | 8.21 | 3.88 (2.61–7.43) |
|  | Middle Pulse Dominant Frequency (kHz) | 1.33 | 0.09 | 1.18–1.44 (1.07–1.46) | 7.00 | 3.70 (1.80–7.04) |
|  | Last Pulse Dominant Frequency (kHz) | 1.34 | 0.12 | 1.10–1.47 (0.94–1.55) | 8.82 | 4.90 (2.23–10.63) |
| Temporal properties of pulse of maximum amplitude | Time of Pulse (ms) from the beginning of call | 294.11 | 56.82 | 221.01–388.54 (156.04–442.71) | 19.32 | 12.03 (5.48–20.22) |
|  | Pulse Period (ms) | 13.21 | 0.70 | 12.13–14.24 (10.57–16.09) | 5.28 | 6.53 (4.73–7.74) |
|  | Pulse Duration (ms) | 12.80 | 0.62 | 11.96–13.86 (9.93–15.70) | 4.85 | 7.15 (5.43–9.85) |
|  | Pulse Rise Time (ms) | 4.72 | 0.37 | 4.01–5.21 (2.69–7.49) | 7.90 | 20.23 (15.81–24.78) |
|  | Pulse 50% Rise Time (ms) | 0.82 | 0.11 | 0.69–1.21 (0.12–3.92) | 13.39 | 55.09 (31.69–113.94) |
|  | Pulse Fall Time (ms) | 8.07 | 0.76 | 7.17–9.41 (5.09–10.34) | 9.45 | 11.23 (4.84–14.50) |
|  | Pulse 50% Fall Time (ms) | 3.66 | 0.84 | 2.64–5.29 (0.15–7.83) | 22.91 | 38.59 (23.04–50.82) |
| Spectral property of pulse of maximum amplitude | Pulse Dominant Frequency (kHz) | 1.39 | 0.11 | 1.18–1.52 (1.07–1.59) | 7.98 | 3.98 (2.44–7.42) |
| * For pulses per call, the values reported in the columns headed 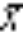and SD, respectively, are the median and interquartile range. Note, however, that coefficient of variation for this call property is computed from 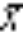and SD. | | | | | | |
