# Supplementary material for: A unique mating strategy without physical contact during fertilization in Bombay Night Frogs (Nyctibatrachus humayuni) with the description of a new form of amplexus and female call
Source: PeerJ. 2016 Jun 14;4:e2117. doi: 10.7717/peerj.2117 (PMC4911947; doi:10.7717/peerj.2117)
Supplement: Supplemental Information 18 — Bold type indicates statistically significant correlation (p < 0.05). [file peerj-04-2117-s018.doc]

Supplemental Information: Table S3

Bert Willaert, Robin Suyesh, Sonali Garg, Varad B Giri, Mark A Bee and SD Biju

A unique mating strategy without physical contact during fertilization in Bombay Night Frog (*Nyctibatrachus humayuni*) with the description of a new form of amplexus and female call

**Table S3 Spearman Rank correlation between mean properties of male *Nyctibatrachus humayuni* calls and SVL, mass and temperature (wet and dry) (n=8).** Bold type indicates statistically significant correlation (p < 0.05).

| Type of acoustic properties | Property | SVL | | Mass |  | Dry bulb temp. (^o^C) | | Wet bulb temp (^o^C) | | Body Condition Index | |
| --- | --- | --- | --- | --- | --- | --- | --- | --- | --- | --- | --- |
|  |  | r_s_ | P | r_s_ | P | r_s_ | P | r_s_ | P | r_s_ | P |
| A. Entire call |  |  |  |  |  |  |  |  |  |  |  |
| Temporal call properties | Call Duration (s) | -0.40 | 0.32 | -0.33 | 0.42 | -0.21 | 0.77 | -0.47 | 0.24 | 0.17 | 0.70 |
|  | Call Rise Time (ms) | -0.16 | 0.69 | 0.24 | 0.57 | -0.14 | 0.73 | -0.40 | 0.31 | 0.00 | 1.00 |
|  | Call Fall Time (ms) | -0.29 | 0.49 | -0.09 | 0.82 | -0.54 | 0.60 | -0.20 | 0.62 | -0.12 | 0.78 |
| Spectral Property | Overall Dominant Frequency (kHz) | **-0.72** | **0.046** | **-0.83** | **0.01** | -0.08 | 0.84 | 0.20 | 0.62 | -0.70 | 0.06 |
| B. First call part |  |  |  |  |  |  |  |  |  |  |  |
| Temporal call properties | Duration 1st part (ms) | 0.09 | 0.82 | -0.21 | 0.61 | -0.26 | 0.52 | -0.46 | 0.24 | -0.12 | 0.78 |
|  | Rise Time (ms) | 0.16 | 0.69 | -0.23 | 0.57 | -0.14 | 0.73 | -0.40 | 0.31 | 0.00 | 1.00 |
|  | Fall Time (ms) | 0.11 | 0.77 | -0.11 | 0.77 | -0.48 | 0.22 | -0.42 | 0.29 | **-0.88** | **0.01** |
| Spectral call Properties | Overall Dominant Frequency (kHz) | **-0.71** | **0.04** | **-0.83** | **0.01** | -0.08 | 0.84 | 0.20 | 0.62 | -0.69 | 0.06 |
|  | Dominant Frequency 1 (kHz) | -0.69 | 0.05 | **-0.83** | **0.01** | -0.12 | 0.77 | 0.22 | 0.58 | -0.64 | 0.09 |
|  | Dominant Frequency 2 (kHz) | **-0.73** | **0.03** | **-0.85** | **0.01** | -0.08 | 0.84 | 0.28 | 0.48 | -0.62 | 0.10 |
|  | Dominant Frequency 3 (kHz) | -0.66 | 0.07 | **-0.76** | **0.02** | -0.19 | 0.64 | 0.20 | 0.62 | -0.64 | 0.09 |
|  | Dominant Frequency 4 (kHz) | **-0.71** | **0.04** | **-0.83** | **0.01** | -0.08 | 0.84 | 0.20 | 0.62 | -0.69 | 0.06 |
| C. Second call part |  |  |  |  |  |  |  |  |  |  |  |
| Temporal call properties | Duration 2nd part (ms) | -0.35 | 0.38 | -0.04 | 0.91 | 0.09 | 0.82 | 0.11 | 0.79 | 0.40 | 0.32 |
|  | Rise Time (ms) | 0.19 | 0.65 | 0.42 | 0.28 | 0.55 | 0.15 | 0.42 | 0.29 | 0.40 | 0.32 |
|  | Fall Time (ms) | -0.42 | 0.28 | -0.14 | 0.73 | 0.01 | 0.97 | 0.00 | 1.00 | 0.29 | 0.49 |
|  | # Pulses per Call (2nd part) | -0.50 | 0.20 | -0.19 | 0.65 | 0.15 | 0.71 | 0.14 | 0.73 | 0.38 | 0.35 |
|  | Pulse Rate (pulses/s) (2nd part) | -0.02 | 0.95 | -0.02 | 0.95 | 0.49 | 0.22 | 0.25 | 0.54 | 0.21 | 0.61 |
| Temporal pulse properties | First Pulse Period (ms) | 0.00 | 1.00 | 0.11 | 0.77 | -0.53 | 0.17 | -0.49 | 0.21 | -0.69 | 0.06 |
|  | Middle Pulse Period (ms) | 0.21 | 0.61 | 0.30 | 0.45 | -0.48 | 0.22 | -0.50 | 0.20 | -0.69 | 0.06 |
|  | "N-1" Pulse Period (ms) | -0.16 | 0.69 | -0.23 | 0.57 | -0.62 | 0.09 | 0.01 | 0.97 | -0.43 | 0.29 |
| Spectral properties | Overall Dominant Frequency 2nd part (kHz) | **-0.71** | **0.04** | **-0.83** | **0.01** | -0.08 | 0.84 | 0.20 | 0.62 | -0.36 | 0.39 |
|  | First Pulse Dominant Frequency (kHz) | **-0.71** | **0.04** | **-0.83** | **0.01** | -0.08 | 0.84 | 0.20 | 0.62 | -0.12 | 0.78 |
|  | Middle Pulse Dominant Frequency (kHz) | -0.61 | 0.10 | **-0.80** | **0.01** | -0.14 | 0.73 | 0.31 | 0.44 | 0.05 | 0.91 |
|  | Last Pulse Dominant Frequency (kHz) | **-0.80** | **0.01** | **-0.92** | **0.01** | -0.05 | 0.90 | 0.30 | 0.46 | -0.07 | 0.87 |
| Temporal properties of pulse of maximum amplitude | Pulse Period (ms) | 0.00 | 1.00 | 0.11 | 0.77 | -0.53 | 0.17 | -0.49 | 0.21 | -0.69 | 0.06 |
|  | Pulse Duration (ms) | 0.02 | 0.95 | 0.14 | 0.73 | -0.44 | 0.26 | -0.38 | 0.34 | -0.12 | 0.78 |
|  | Pulse Rise Time (ms) | 0.09 | 0.82 | 0.07 | 0.86 | 0.14 | 0.73 | -0.34 | 0.39 | -0.05 | 0.91 |
|  | Pulse 50% Rise Time (ms) | 0.66 | 0.07 | **0.83** | **0.01** | -0.15 | 0.71 | -0.39 | 0.32 | 0.21 | 0.61 |
|  | Pulse Fall Time (ms) | -0.11 | 0.77 | -0.02 | 0.95 | -0.53 | 0.17 | -0.24 | 0.56 | 0.26 | 0.53 |
|  | Pulse 50% Fall Time (ms) | 0.14 | 0.73 | 0.71 | 0.86 | -0.42 | 0.29 | 0.22 | 0.58 | -0.19 | 0.65 |
| Spectral property of pulse of maximum amplitude | Pulse Dominant Frequency (kHz) | **-0.71** | **0.04** | **-0.83** | **0.01** | -0.08 | 0.84 | 0.20 | 0.62 | -0.26 | 0.53 |
