# Supplementary material for: A unique mating strategy without physical contact during fertilization in Bombay Night Frogs (Nyctibatrachus humayuni) with the description of a new form of amplexus and female call
Source: PeerJ. 2016 Jun 14;4:e2117. doi: 10.7717/peerj.2117 (PMC4911947; doi:10.7717/peerj.2117)
Supplement: Supplemental Information 19 [file peerj-04-2117-s019.doc]

Supplemental Information: Table S4

Bert Willaert, Robin Suyesh, Sonali Garg, Varad B Giri, Mark A Bee and SD Biju

A unique mating strategy without physical contact during fertilization in Bombay Night Frog (*Nyctibatrachus humayuni*) with the description of a new form of amplexus and female call

**Table S4 Snout-Vent Length (SVL) and body mass of recorded male individuals, with corresponding dry bulb and wet bulb temperature.**

| # Male | SVL (mm) | Body mass (g) | Dry bulb  temperature (^o^C) | Wet bulb  temperature (^o^C) |
| --- | --- | --- | --- | --- |
| 1 | 40.5 | 6.37 | 22.2 | 23.3 |
| 2 | 46.1 | 9.18 | 22.5 | 23.2 |
| 3 | 50.1 | 11.99 | 22.5 | 23.2 |
| 4 | 51.9 | 12.78 | 21.6 | 22.5 |
| 5 | 45.6 | 8.22 | 21.8 | 22.3 |
| 6 | 45.2 | 11.26 | 23.5 | 22.8 |
| 7 | 43.9 | 7.66 | 22.2 | 22.8 |
| 8 | 48.1 | 11.45 | 21.4 | 22.2 |
