# Supplementary material for: A unique mating strategy without physical contact during fertilization in Bombay Night Frogs (Nyctibatrachus humayuni) with the description of a new form of amplexus and female call
Source: PeerJ. 2016 Jun 14;4:e2117. doi: 10.7717/peerj.2117 (PMC4911947; doi:10.7717/peerj.2117)
Supplement: Supplemental Information 20 — Shown here are the means (X), standard deviation (SD) and range of minimum and maximum values across all analyzed calls. CVs computed within individuals (CVw). [file peerj-04-2117-s020.doc]

Supplemental Information: Table S5

Bert Willaert, Robin Suyesh, Sonali Garg, Varad B Giri, Mark A Bee and SD Biju

A unique mating strategy without physical contact during fertilization in Bombay Night Frog (*Nyctibatrachus humayuni*) with the description of a new form of amplexus and female call

**Table S5 Descriptive statistics of entire call of female *Nyctibatrachus humayuni* based on the values determined from a sample of 1 individual (20 calls).** Shown here are the means (
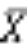
), standard deviation (SD) and range of minimum and maximum values across all analyzed calls. CVs computed within individuals (CVw).

| **Type of acoustic properties** | **Property** | 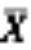 | **SD** | **Range (min-max)** | **Mean CV_W_ (range)** |
| --- | --- | --- | --- | --- | --- |
| Temporal call properties | Call Duration (ms) | 83.77 | 15.33 | 40.73–112.60 | 18.30 |
|  | Call Rise Time (ms) | 16.75 | 4.67 | 7.58–29.38 | 27.90 |
|  | Call 50% rise time (ms) | 6.14 | 3.56 | 0.83–17.57 | 58.04 |
|  | Call Fall Time (ms) | 66.99 | 15.38 | 20.38–84.16 | 22.96 |
|  | Call 50 % fall time | 32.31 | 14.48 | 9.27–60.42 | 44.81 |
| Spectral properties | Overall Dominant Frequency (kHz) | 2.85 | 0.35 | 1.39–3.10 | 12.37 |
|  | Overall Dominant Frequency 1 (kHz) | 1.45 | 0.04 | 1.39–1.55 | 3.16 |
|  | Overall Dominant Frequency 2 (kHz) | 2.91 | 0.16 | 2.50–3.05 | 3.98 |
|  | Overall Dominant Frequency 3 (kHz) | 4.37 | 0.11 | 4.13–4.52 | 2.53 |
