# Supplementary material for: A unique mating strategy without physical contact during fertilization in Bombay Night Frogs (Nyctibatrachus humayuni) with the description of a new form of amplexus and female call
Source: PeerJ. 2016 Jun 14;4:e2117. doi: 10.7717/peerj.2117 (PMC4911947; doi:10.7717/peerj.2117)
Supplement: Supplemental Information 21 — The column ‘species’ corresponds to the species’ name as used in the corresponding reference. The columns ‘current species’ and ‘family’ correspond to present-day taxonomy (Frost, 2015). [file peerj-04-2117-s021.doc]

Supplemental Information: Table S6

Bert Willaert, Robin Suyesh, Sonali Garg, Varad B Giri, Mark A Bee and SD Biju

A unique mating strategy without physical contact during fertilization in Bombay Night Frog (*Nyctibatrachus humayuni*) with the description of a new form of amplexus and female call

**Table S6 Overview of anuran species in which a female call has been described.** The column ‘species’ corresponds to the species’ name as used in the corresponding reference. The columns ‘current species’ and ‘family’ correspond to present-day taxonomy (*Frost 2015*).

| **Species** | **Current species** | **Family** | **Reference** |
| --- | --- | --- | --- |
| *Alytes cisternasii* | *Alytes cisternasii* | Alytidae | *Bosch & Márquez, 2001* |
| *Alytes muletensis* | *Alytes muletensis* | Alytidae | *Bush, Dyson & Halliday, 1996* |
| *Alytes obstetricans* | *Alytes obstetricans* | Alytidae | *Heinzmann, 1970* |
| *Bombina variegata* | *Bombina variegata* | Bombinatoridae | *Savage, 1932* |
| *Platymantis vitiensis* | *Platymantis vitiensis* | Ceratobatrachidae | *Boistel & Sueur, 1997* |
| *Ceratobatrachus guentheri* | *Cornufer guentheri* | Ceratobatrachidae | *Yoshimi et al., 1996* |
| *Eleutherodactylus podiciferus* | *Craugastor podiciferus* | Craugastoridae | *Schlaepfer & Figeroa-sandi, 1998* |
| *Rana blythi* | *Limnonectes leporinus* | Dicroglossidae | *Emerson, 1992* |
| *Rana cyanophlyctis* | *Euphlyctis cyanophlyctis* | Dicroglossidae | *Roy, Borah & Sarma, 1995* |
| *Rana limnocharis* | *Fejervarya limnocharis* | Dicroglossidae | *Roy, Borah & Sarma, 1995* |
| *Eleutherodactylus angustidigitorum* | *Eleutherodactylus angustidigitorum* | Eleutherodactylidae | *Dixon, 1957* |
| *Eleutherodactylus guanahacabibes* | *Eleutherodactylus guanahacabibes* | Eleutherodactylidae | *Diáz & Estrada, 2000* |
| *Hyla microcephala* | *Dendropsophus microcephalus* | Hylidae | *Schwartz J, personal communication in Wells, 2007* |
| *Leptodactylus fragilis* | *Leptodactylus fragilis* | Leptodactylidae | *Bernal & Ron, 2004* |
| *Leptodactylus syphax* | *Leptodactylus syphax* | Leptodactylidae | *Da Silva, Giaretta & Facure, 2008* |
| ***Nyctibatrachus humayuni*** | ***Nyctibatrachus humayuni*** | **Nyctibatrachidae** | **Present study** |
| *Pelobates cultripes* | *Pelobates cultripes* | Pelobatidae | *Lizana, Marquez & Martin-Sanchez, 1994* |
| *Pelobates fuscus* | *Pelobates fuscus* | Pelobatidae | *Andreone & Piazza, 1990* |
| *Xenopus laevis* | *Xenopus laevis* | Pipidae | *Tobias, Viswanathan & Kelley, 1998* |
| *Babina daunchina* | *Babina daunchina* | Ranidae | *Cui et al., 2010* |
| *Rana catesbeiana* | *Lithobates catesbeianus* | Ranidae | *Judge, Swanson & Brooks, 2000* |
| *Rana curtipes* | *Clinotarsus curtipes* | Ranidae | *Krishna & Krishna, 2005* |
| *Rana erythraea* | *Hylarana erythraea* | Ranidae | *Roy, Borah & Sarma, 1995* |
| *Rana ridibunda* | *Pelophylax ridibundus* | Ranidae | *Frazer, 1983* |
| *Rana virgatipes* | *Lithobates virgatipes* | Ranidae | *Given, 1987* |

REFERENCES

Andreone F, Piazza R. 1990. A bioacoustic study on *Pelobates fuscus insubricus* (Amphibia, Pelobatidae). *Bolletino di zoologia* **57**:341–349 DOI 10.1080/11250009009355717.

Bernal X, Ron SR. 2004. *Leptodactylus fragilis* (White-lipped Foam frog). Courtship. *Herpetological Review* **35**:372–373.

Boistel R, Sueur J. 1997. Comportement sonore de la femelle de *Platymantis vitiensis* (Amphibia , Anura) en l’absence du mâle. *Comptes Rendus l’Académie des Sci - Ser III - Sci la Vie*. **320**:933–941 DOI 10.1016/S0764-4469(97)80879-7.

Bosch J, Márquez R. 2001. Female courtship call of the Iberian Midwife Toad (*Alytes cisternasii*). *Journal of Herpetology* **35**:647–652 DOI 10.2307/1565904.

Bush SL, Dyson ML, Halliday TR. 1996. Selective phonotaxis by males in the Majorcan midwife toad. *Proceedings of the Royal Society B, London* **263**:913–917 DOI 10.1098/rspb.1996.0135.

Cui J, Wang Y, Brauth S, Tang Y. 2010. A novel female call incites male-female interaction and male-male competition in the Emei music frog, *Babina daunchina*. *Animal behaviour* **80**:181–187 DOI 10.1016/j.anbehav.2010.05.012.

Da Silva WR, Giaretta AA, Facure KG. 2008. Vocal repertory of two species of the *Leptodactylus pentadactylus* group (Anura, Leptodactylidae). *Contemporary Herpetology* **2008**:1–6.

Diáz LM, Estrada AR. 2000. The male and female vocalizations of the Cuban Frog *Eleutherodactylus guanahacabibes* (Anura: Leptodactylidae). *Caribbean Journal of Science* **36**:328–331.

Dixon JR. 1957. Geographic variation and distribution of the genus *Tomodactylus* in Mexico.Texas: A&M University.

Emerson SB. 1992. Courtship and vest-building behavior of a Bornean frog , *Rana blythi. Copeia* **1992**:1123–1127.

Frazer D. 1983. *Reptiles and Amphibians of Britain*. Glasgow: William Collins Sons & Co. Ltd.

Given MF. 1987. Vocalizations and Acoustic Interactions of the Carpenter Frog, *Rana virgatipes*. *Herpetologica* **43**:467–481.

Heinzmann U. 1970. Untersuchungen zur Bio-Akustik und ökologie der Geburtshelferkröte, *Alytes o. obstetricans* (Laur.). *Oecologia* **5**:19–55 DOI 10.1007/BF00345974.

Judge KA, Swanson SJ, Brooks RJ. 2000. *Rana catesbeiana* (Bullfrog). female vocalization. *Herpetological Review* **31**:236–237.

Krishna S, Krishna S. 2005. Female courtship calls of the litter frog (*Rana curtipes*) in the tropical forests of Western Ghats, South India. *Amphibia-Reptilia* **26**:431–435 DOI 10.1163/156853805774806179.

Lizana M, Marquez R, Martin-Sanchez R. 1994. Reproductive biology of *Pelobates cultripes* (Anura: Pelobatidae) in Central Spain. *Journal of Herpetology* **28**:19–27 DOI 10.2307/1564675.

Roy D, Borah B, Sarma A. 1995. Analysis and significance of female reciprocal call in frogs. *Current Science* **69**:265–270.

Savage RM. 1932. The spawning, voice, and sexual behaviour of *Bombina variegata* variegata. *Journal of Zoology*  **4**:889–898 DOI 10.1111/j.1096-3642.1932.tb01570.x

Schlaepfer MA, Figeroa-sandi R. 1998. Female Reciprocal Calling in a Costa Rican Leaf-Litter Frog, *Eleutherodactylus podiciferus*. Copeia **1998**:1076–1080 DOI 10.2307/1447362.

Tobias ML, Viswanathan SS, Kelley DB. 1998. Rapping, a female receptive call, initiates male-female duets in the South African clawed frog. *Proceedings of the National Academy of Sciences of the United States of America* **95**:1870–1875 DOI 10.1073/pnas.95.4.1870.

Wells KD. 2007. *The Ecology and Behaviour of Amphibians*. Chicago: The University of Chicago Press.

Yoshimi DH, Payne DA, Slavens FL. 1996. Maintenance and Captive Breeding of the Solomon Islands Leaf Frog (*Ceratobatrachus guentheri*). In: Strimple PD, ed. *Advances in Herpetoculture*. International Herpetological Symposium, Inc., 23–32.
